# An mRNA Vaccine Expressing Blood-Stage Malaria Antigens Induces Complete Protection Against Lethal *Plasmodium yoelii*

**DOI:** 10.3390/vaccines13070702

**Published:** 2025-06-28

**Authors:** Amy C. Ott, Patrick J. Loll, James M. Burns

**Affiliations:** 1 Center for Molecular Parasitology, Department of Microbiology and Immunology, Drexel University College of Medicine, 2900 West Queen Lane, Philadelphia, PA 19129, USA; jmb53@drexel.edu; 2 Department of Biochemistry and Molecular Biology, Drexel University College of Medicine, 215 North 15th St., Philadelphia, PA 19102, USA; pjl28@drexel.edu

**Keywords:** malaria, *Plasmodium*, *Plasmodium yoelii*, vaccines, mRNA, blood-stage malaria

## Abstract

Background and Objectives: To evaluate the mRNA vaccine platform for blood-stage *Plasmodium* parasites, we completed a proof-of-concept study using the *P. yoelii* mouse model of malaria and two mRNA-based vaccines. Both encoded *Py*MSP1_19_ fused to *Py*MSP8 (*Py*MSP1/8). One was designed for secretion of the encoded protein (*Py*MSP1/8-sec); the other encoded membrane-bound antigen (*Py*MSP1/8-mem). Methods: Secretion of *Py*MSP1/8-sec and membrane localization of *Py*MSP1/8-mem were verified in mRNA-transfected cells. As recombinant *Py*MSP1/8 (r*Py*MSP1/8) is known to protect mice against lethal *P. yoelii* 17XL infection, we first compared immunogenicity and efficacy of the *Py*MSP1/8-sec mRNA vaccine versus the recombinant formulation in outbred mice. Animals were immunized three times followed by challenge with a lethal dose of *P. yoelii* 17XL-parasitized RBCs (pRBCs). Similar immunization and challenge experiments were conducted to compare *Py*MSP1/8-sec versus *Py*MSP1/8-mem mRNA vaccines. Results: Immunogenicity of the *Py*MSP1/8-sec mRNA vaccine was superior to the recombinant formulation, inducing higher antibody titers against both vaccine components. Following challenge with *P. yoelii* 17XL pRBCs, all *Py*MSP1/8-sec-immunized animals survived, with 50% of these showing no detectible pRBCs in circulation (<0.01%). In addition, mean peak parasitemia in *Py*MSP1/8-sec mRNA-immunized mice was significantly lower than that in the r*Py*MSP1/8 vaccine group. Both *Py*MSP1/8-sec and *Py*MSP1/8-mem were protective against *P. yoelii* 17XL challenge, with *Py*MSP1/8-mem immunization providing a significantly higher level of protection than *Py*MSP1/8-sec immunization considering the number of animals with no detectable pRBCs in circulation and the mean peak parasitemia in animals with detectable parasitemia. Conclusions: mRNA vaccines were highly immunogenic and potently protective against blood-stage malaria, outperforming a similar recombinant-based vaccine. The membrane-bound antigen was more effective at inducing protective antibody responses, highlighting the need to consider antigen localization for mRNA vaccine design.

## 1. Introduction

Worldwide, there were an estimated 597,000 deaths due to malaria in 2023, with the majority of these in children under the age of five [1]. Morbidity and mortality are predominantly due to infection with *Plasmodium falciparum*, one of five species that cause human malaria [2]. Innovative drug treatments, vector control strategies, and effective vaccines are urgently needed, especially for young children living in endemic areas.

*Plasmodium* parasites complete a complex life cycle involving a mammalian host and a female *Anopheles* mosquito vector. Current vaccine strategies focus on targeting pre-erythrocytic and blood-stage parasites as well as blocking transmission of sexual-stage parasites from the human host to the mosquito vector [3]. The recent approval of RTS,S/AS01 and R21/Matrix-M malaria vaccines, which target pre-erythrocytic stage parasites, represents a significant and important milestone in malaria vaccine development. Both vaccines are recombinant antigen-based formulations targeting *P. falciparum* circumsporozoite protein (*Pf*CSP) fused to hepatitis B surface antigen [4,5]. A phase III trial in African children immunized with R21/Matrix-M indicated a 12-month efficacy (i.e., time to first malaria episode) of ~75%, a significant improvement over RTS,S/AS01 [6]. However, because RTS,S and R21 target a single pre-erythrocytic-stage malarial antigen, vaccinated children remain fully susceptible to blood-stage infection when sporozoites escape neutralization. Therefore, the current *Pf*CSP-based vaccines targeting the pre-erythrocytic stage would benefit from inclusion of one or more blood-stage antigens [3,4,7].

The overall goal of blood-stage malaria vaccine development is to generate high-titer antibodies that block merozoite invasion of host RBCs and/or target parasite antigens expressed on the surface of infected RBCs [8,9]. The large number of merozoites that are extracellular for only a short period of time; the ability of merozoites to utilize multiple, distinct pathways for invasion of RBCs; and the polymorphism of many vaccine candidate antigens present challenges [10]. However, early passive transfer experiments showed that immune sera can clear parasites in susceptible individuals, highlighting the importance of antibodies during this stage of infection [11]. *P. falciparum* blood-stage vaccine candidates include merozoite surface proteins (MSPs), erythrocyte-binding antigen-175 (EBA-175), apical membrane antigen-1 (AMA-1), glutamate-rich protein (GARP), serine repeat antigen 5 (SERA5), erythrocyte membrane protein-1 (EMP-1), and reticulocyte-binding protein homologue 5 (Rh5) [3,7,8,10]. The most recent clinical trials for blood-stage malaria vaccines focus on *Pf*Rh5. A Phase 1b study in healthy Tanzanian adults and children immunized with *Pf*Rh5 recombinant antigen formulated with Matrix M as adjuvant demonstrated encouraging results with respect to safety and immunogenicity [12].

In general, recombinant protein-based malaria vaccines have faced challenges associated with proper expression and folding of target antigens, large-scale production, and low immunogenicity [13]. The recent widespread success of the SARS-CoV-2 mRNA vaccines in both children and adults [14,15,16,17] highlight the enormous potential of the mRNA platform. With improvements in construct design, the incorporation of modified nucleosides, and the use of lipid nanoparticles (LNPs) for delivery [18], the mRNA platform provides significant advantages over recombinant antigen formulations, including ease of production, scalability, and improved immunogenicity. LNPs also allow targeted delivery to specific organs or cells [19] to broadly elicit strong CD4+ and CD8+ T-cell responses while concurrently driving production of high-titer neutralizing antibodies [19].

The list of mRNA-based vaccines is growing, largely targeting viral pathogens [20]. However, a few studies have begun to evaluate mRNA-based vaccines for *P. falciparum* antigens [21,22,23,24,25,26], but the platform needs to be more fully evaluated and optimized to address issues unique to *Plasmodium* and malaria. With mRNA-based malaria vaccines, the efficiency of antigen expression in host cells, folding, and secretion need consideration. Differences in mammalian versus plasmodial post-translational modifications also need attention. Where and when a vaccine antigen is expressed, processed, and presented in vivo will impact immunogenicity. For example, the SARS CoV2 BNT162b2 mRNA vaccine encoding the native spike protein with its transmembrane domain intact was chosen over a similar construct targeting the spike protein for secretion, in response to consideration of both safety and immunogenicity [27]. Membrane-bound antigen may prolong protein accessibility in vivo, influence antigen uptake and processing by APCs, increase B-cell receptor cross-linking to improve quality and quantity of antigen-specific antibodies, and/or improve durability of protection [28,29,30].

*Plasmodium* merozoite surface protein-1 (MSP1) is a surface-expressed, glycosylphosphatidylinositol (GPI)-anchored membrane protein. MSP1 is an essential, conserved blood-stage antigen that is proteolytically processed into multiple fragments, including a 19 kDa C-terminal domain (MSP1_19_), expressed on the merozoite surface [31,32,33]. Two epidermal growth factor (EGF)-like domains within MSP1_19_ are the target of neutralizing antibodies, but T-cell recognition of this region is weak [34,35,36,37,38]. Merozoite surface protein-8 (MSP8) is a second GPI-anchored protein with two C-terminal EGF-like domains, similar to the protective EGF-like domains of MSP1_19_ [39]. Mice immunized with r*Py*MSP8 are protected against lethal *P. yoelii* 17XL malaria [40]. Protection induced by r*Py*MSP8 is B-cell-dependent, requiring induction of antibodies that recognize disulfide-dependent epitopes. Protective antibodies are independent of IgG subclass but require both IFNγ and IL-4 for a maximal induction [41]. We previously demonstrated that fusion of recombinant MSP1_19_ to merozoite surface protein-8 (MSP8) elicited strong CD4+ T-cell help for production of merozoite neutralizing antibodies [42]. Immunization with recombinant *Plasmodium yoelii* MSP1/8 (r*Py*MSP1/8) formulated with Quil A as adjuvant afforded enhanced protection against *P. yoelii* 17XL infection, relative to individual r*Py*MSP1 or r*Py*MSP8 vaccine formulations [43].

To evaluate the potential of the mRNA platform for blood-stage malaria vaccines, we carried out a proof-of-concept study using the *P. yoelii* mouse model of malaria. We developed two related mRNA-based vaccine constructs encoding *Py*MSP1_19_ fused to *Py*MSP8 (*Py*MSP1/8). One construct, *Py*MSP1/8-sec, was designed for secretion of the expressed protein, while the second encoded membrane-bound protein (*Py*MSP1/8-mem). We confirmed *Py*MSP1/8 expression in mRNA-transfected mammalian cells and verified proper secretion versus membrane localization. To determine if the mRNA-based vaccine platform is effective for immunization against blood-stage malaria parasites, we compared the immunogenicity and in vivo efficacy of these two *Py*MSP1/8 mRNA/LNP vaccines relative to the previously described and potently protective recombinant antigen plus adjuvant r*Py*MSP1/8 vaccine.

## 2. Materials and Methods

### 2.1. mRNA Vaccine Construct Design

A DNA template including a T7 promotor, 5′ UTR from human alpha globin, and the coding sequence from a mouse MHC class II beta-chain signal sequence was fused to a *Py*MSP1/8 synthetic gene, which was codon-harmonized for expression in mice [44]. This was followed by a 3′ UTR derived from amino-terminal enhancer of split (AES) mRNA, the mitochondrial 12S ribosomal RNA sequence, and a >75 nucleotide poly A tail. Three glycosylation sites were modified (N to Q) to avoid detrimental N-linked glycosylation of the double EGF-like domains of MSP1_19_ [45]. In addition, the *Py*MSP1/8-mem construct includes the C-terminal signal sequence from mouse CD14, which is required for the addition of a GPI anchor and surface expression [46]. Coding sequences were confirmed by sequencing (GenBank Accession #PV599979 and #PV599980).

### 2.2. Production of Capped, Purified mRNA, and CHO Cell Transfection

Messenger RNA was transcribed in vitro (T7 Megascript Kit, Thermo Fisher Scientific, Inc., Waltham, MA, USA) by replacing UTP with N1-methylpseudouridine-5′-triphosphate (TriLink Biotechnologies, San Diego, CA, USA) and was then capped (Cellscript, Madison, WI, USA) and purified. Contaminated double-stranded RNA was removed using a cellulose column as described [47]. Agarose gel electrophoresis confirmed that transcripts were of the correct size (RNA Millenium Marker, Invitrogen by ThermoFisher, Waltham, MA, USA). For mRNA transfection studies, Chinese hamster ovary (CHO) K1 cells (American Type Culture Collection, Manassas, VA, USA) were maintained in F12K media plus 10% heat-inactivated fetal bovine serum (FBS), 100 µg/mL streptomycin, and 100 U/mL penicillin. Trypsin—0.05%/EDTA—0.53 mM was used for serial passage. Cells were resuspended to 3 × 10^5^ cells/mL and plated into 24-well tissue culture plates for immunoblot assays or 4-well chamber slides for immunofluorescence assays (Nunc™ Lab-Tek Chamber Slides, Thermo Fisher Scientific, Waltham, MA, USA) and incubated at 37 °C/5% CO_2_ for 24 h. Cells were transfected with 1 µg per well of capped, purified mRNA according to manufacturer’s protocol (*Trans*-IT^®^ mRNA Transfection Kit, Mirus Bio, Madison, WI, USA) and harvested after 24, 48, or 72 h. *Xenopus* elongation factor 1α (Xef) mRNA-transfected cells were used as a negative control.

### 2.3. Immunoblot Assays

Following CHO cell transfections, cell supernatants were collected. Cell-associated fractions were isolated by adding 0.53 mM ETDA in 1× phosphate-buffered saline (PBS) to culture wells followed by centrifugation. Samples were separated on a 10% SDS-PAGE gel in the presence of 5% beta-mercaptoethanol, followed by analysis using rabbit sera (diluted 1:5000) recognizing *P. yoelii* MSP8, as previously described [48]. Bound antibody was detected using a goat anti-rabbit IgG conjugated to horseradish peroxidase (HRP) (Invitrogen, Carlsbad, CA, USA) diluted 1:5000 followed by SuperSignal™ West Pico PLUS Chemiluminescent Substrate (Thermo Fisher Scientific). Immunoblots were visualized using a Chemidoc imaging system (Bio-Rad Laboratories, Inc., Hercules, CA, USA).

### 2.4. Immunofluorescence Assay

Approximately 48 h following transfection, cells were fixed in 1× PBS containing 4% formaldehyde and 0.0075% glutaraldehyde. A second set of cells was fixed as above and permeabilized with 0.1% Triton X-100. To reduce background fluorescence, cells were incubated with 0.1 mg/mL NaBH_4_, washed, and blocked with 5% non-fat dry milk in 1× PBS. Rabbit anti-*Py*MSP8 sera was diluted 1:500, added to the chamber slides, and incubated for one hour at room temperature. Bound antibody was detected with fluorescein isothiocyanate (FITC)-conjugated goat anti-rabbit IgG (Thermo Fisher Scientific) diluted 1:400. Between each step, cells were washed three times with 1× PBS. Finally, cells were stained with 4′, 6-Diamino-2-phenylindole dihydrochloride (DAPI) plus Antifade solution (Sigma-Aldrich, Inc., St. Louis, MO, USA) prior to visualization. Images were acquired using an Olympus fluorescence microscope (Evident Scientific, Waltham, MA, USA) and processed using NIS Elements AR software (Version 5.30.02, Nikon Instruments, Inc., Melville, NY, USA).

### 2.5. Production of mRNA-LNP Vaccines

Messenger RNA was encapsulated into lipid nanoparticlcontaining (i) an ionizable lipid, ((4-hydroxybutyl)azanediyl)bis(hexane-6,1-diyl)bis(2-hexyldecanoate)), (ii) two structural lipids (1,2-distearoyl-sn-glycero-3-phosphocholine (DSPC) and cholesterol), and (iii) a PEGylated lipid (2-[(polyethylene glycol)-2000]-*N*,*N*-ditetradecylacetamide) (lipid molar ratios—46.3:9.4:42.7:1.6 mol%), according to manufacturer’s instructions (Lipid Launch™ LNP-0315 lipid exploration kit; Cayman Chemical, Ann Arbor, MI, USA), with minor modifications: a 1:20 RNA-to-lipid ratio and a 1:3 lipid-volume-to-aqueous-volume ratio were maintained, and mRNA was prepared in 50 mM sodium acetate pH 4. mRNA-LNPs were extruded (Avanti Research, Alabaster, AL, USA) to generate particles of uniform 0.1 µm size. Following overnight dialysis into 1× PBS + 10% sucrose, RNA concentration and percent encapsulation efficiency were calculated using the Quant-it™ RiboGreen RNA assay kit (Thermo Fisher Scientific). To eliminate storage and stability as a variable, LNPs with encapsulated mRNAs were prepared fresh for each immunization. To measure zeta potential, mRNA/LNPs in 1× PBS were concentrated using a 10 kDa molecular weight centrifugal concentrator and subsequently diluted 15-fold with nuclease-free water. Final concentrations were 370 µg/mL (lipid) and 19 µg/mL (RNA), with a final pH of 7.8. Zeta potential was measured in a ZetaSizer Nano (Malvern Pananalytical, Westborough, MA, USA) per the manufacturer’s instructions. Values from three runs were averaged to yield a mean value of −8.7 ± 0.6 mV.

### 2.6. Immunization and Challenge

All animal studies were performed in accordance with protocols approved by Drexel University’s Institutional Animal Care and Use Committee (IACUC; Protocols LA-22-011 and LA-24-067). Animals were housed in pathogen-free conditions in the Animal Care Facility of Drexel University College of Medicine, Queen Lane campus. Male and female 5–6-week-old CD1 mice were purchased from Charles River Laboratories (Wilmington, MA, USA) and were immunized at 6–8 weeks of age. Ten animals per group (five females and five males) were immunized intramuscularly (i.m.) three times at a 3-week interval with *Py*MSP1/8 mRNA/LNPs (1–5 µg per dose as indicated). Selection of the vaccine dose was considered using prior mRNA/LNP vaccine studies in mice, where dose per immunization ranged from 0.2 µg to 30 µg [21,22,49]. Control mice received mRNA/LNPs encoding the Xef. For comparison, groups of mice (*n* = 10, 5F, 5M) were immunized subcutaneously (s.c.) with recombinant *Py*MSP1/8 (5 µg/dose) formulated with Quil A (25 µg/dose) as adjuvant or with adjuvant alone. Antigen/adjuvant doses were based on prior efficacy studies with r*Py*MSP1/8 expressed and purified using nickel-chelate affinity chromatography, as described [43]. All immunized animals were monitored daily. Vaccines were well-tolerated with no local reactions at the site of infection. No changes in animal feeding, hydration, or behavior were observed. Two weeks following the third immunization, animals were challenged by intraperitoneal (i.p.) injection of 1 × 10^6^ *P. yoelii* 17XL-parasitized RBCs. Parasitemia was enumerated by microscopy of thin tail-blood smears stained with Giemsa, with a limit of detection (LOD) of 0.01%. Animals with blood parasitemia greater than 50% and/or those with greater than 10% weight loss were sacrificed and infections scored as lethal.

### 2.7. ELISA

Approximately two weeks following each immunization, a small amount of tail blood was obtained to assess anti-*Py*MSP1_19_ and anti-*Py*MSP8 antibody responses in vaccinated animals, as previously described [43]. Plates were coated with recombinant GST-*Py*MSP1_19_ or *Py*MSP8 (0.25 μg/well) and blocked with 5% nonfat dry milk in 1× TBS (25 mM Tris-HCl, pH 8.0, and 150 mM NaCl). Wells were incubated (2 h at room temperature) with two-fold serial dilutions of individual mouse sera. After washing, bound antibody was detected using a rabbit anti-mouse IgG (H+L) conjugated to HRP (Invitrogen) with ABTS [2,2′-azino-bis(ethylbenzothiazoline-6-sulfonic acid) diammonium salt] as substrate (KPL ABTS Peroxidase Substrate System, Sera Care, Milford, MA, USA). Absorbance was read at 405 nm. To normalize values across plates, a pool of high-titer immunization sera was included. For each sample, the serum dilution factor was plotted against OD_405_ values between 0.1 and 1.0. The titer was then calculated as the reciprocal of the dilution, yielding an OD_405_ of 0.5. To measure IgG subclass, serum samples were titered on recombinant *Py*MSP1/8-coated ELISA plates. Plates were treated as described above, and bound antibodies were detected using HRP-conjugated goat antibodies specific for mouse IgG1, IgG2a, and IgG2c (Southern Biotech, Birmingham, AL, USA), with ABTS as substrate. Each plate also contained wells coated with serial dilutions of an IgG subclass-specific mouse myeloma protein to generate a standard curve. IgG subclass concentrations are expressed as units/mL, with one unit/mL equivalent to 1 µg/mL of myeloma standard.

### 2.8. Statistical Analysis

Statistical analysis was performed using GraphPad Prism v10.1.2 (GraphPad Software Inc., Boston, MA, USA). In all cases, probability (*p*) values less than 0.05 were considered significant. The Mann–Whitney non-parametric test was used to analyze differences in antibody titers between two immunization groups. The Wilcoxon matched-pairs signed-rank test was used to analyze boosting of antibody responses in paired samples after one or two immunizations. Following *P. yoelii* 17XL challenge infection, differences in survival were analyzed using the Mantel–Cox log-rank test.

### 2.9. Generative Artificial Intelligence

GenAI tools were not used in any aspect of the design, execution, analysis, or interpretation of this study and associated data. GenAI tools were not used in the preparation of the manuscript text or graphics.

## 3. Results

### 3.1. PyMSP1/8-Sec and PyMSP1/8-Mem mRNAs Are Expressed in Mammalian Cells

**Figure 1:**
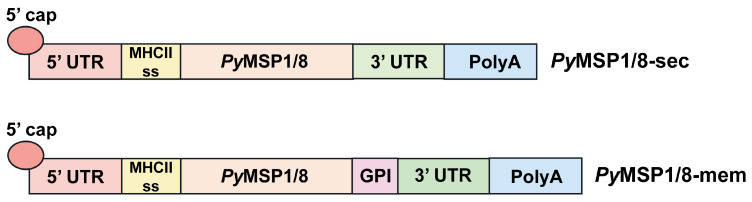
*Py*MSP1/8 mRNA vaccines. Schematic of the key sequence elements incorporated into the *Py*MSP1/8-sec and *Py*MSP1/8-mem mRNA vaccines. 5′ untranslated region (UTR), MHC class II signal sequence, *Py*MSP1/8 coding region, 3′ UTR, and poly A tail are indicated. The glycosylphosphatidylinositol (GPI) anchor signal sequence inserted into the *Py*MSP1/8-mem vaccine construct is noted.

Using the *P. yoelii* mouse model of malaria, two mRNA-based vaccines encoding *Py*MSP1_19_ fused to *Py*MSP8 (*Py*MSP1/8) were developed. The first construct, *Py*MSP1/8-sec was targeted for secretion. The second construct, *Py*MSP1/8-mem was targeted to be anchored on the cell surface membrane. The mRNA construct design paralleled that used for the Pfizer BNT162b2 vaccine, with minor modifications [49]. As diagrammed in Figure 1, the DNA template for both constructs included (i) a T7 promotor, (ii) the 5′ UTR from human α globin, (iii) the coding sequence for a mouse MHC class II signal sequence fused to a *Py*MSP1/8 synthetic gene codon-harmonized for expression in mice, (iv) a 3′ UTR derived from amino-terminal enhancer of split (AES) mRNA and mitochondrial 12S ribosomal sequence, and (v) a 75–80 nucleotide poly A tail. Three N-linked glycosylation sites were modified in the coding sequence (N to Q). To target *Py*MSP1/8-mem for surface display on host cells, the sequence encoding the C-terminal signal sequence from mouse CD14, required for addition of a GPI-anchor, was fused in frame to the 3′ end of the *Py*MSP1/8 coding region. CD14 is a GPI-anchored protein expressed on the surface of dendritic cells and macrophages that binds the LPS-LPS binding protein complex [46].

**Figure 2:**
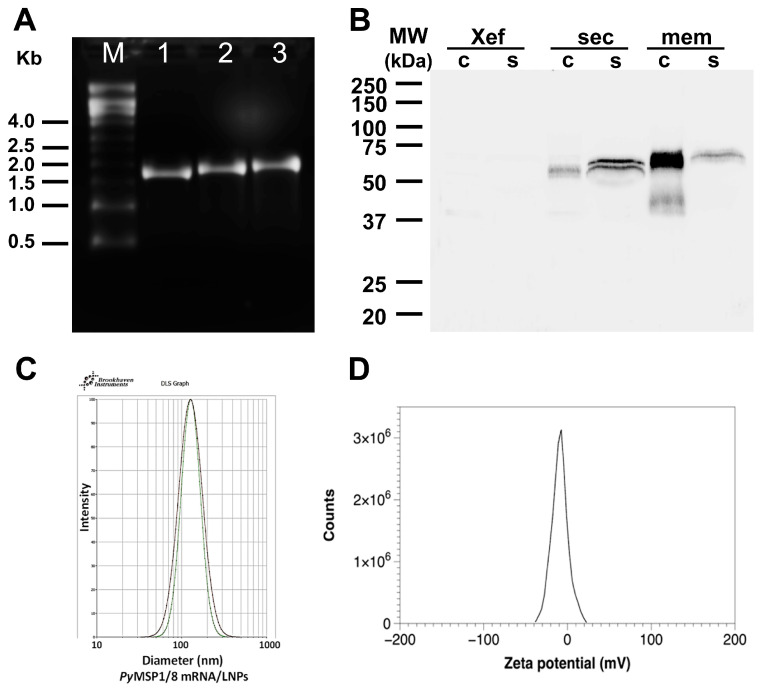
Characterization of *Py*MSP1/8 mRNA vaccines. (**A**) Agarose gel electrophoresis of in vitro transcribed Xef (lane 1), *Py*MSP1/8-sec (lane 2), and *Py*MSP1/8-mem (lane 3) mRNAs. Molecular weight (M) markers in kilobases (kb) are indicated. (**B**) Immunoblot analysis of CHO cell culture supernatant (s) and cells (c) 48 h after transfection with Xef, *Py*MSP1/8-sec, and *Py*MSP1/8-mem mRNA. Proteins were detected using polyclonal anti-*Py*MSP8 antisera. Molecular weight (MW) markers in kilodaltons (kDa) are indicated. (**C**) Representative dynamic light scattering profiles of two *Py*MSP1/8 mRNA-LNP preparations. Diameter in nm is indicated on the *X*-axis. (**D**) Representative zeta potential distribution at pH 7.8, showing a near-neutral surface charge distribution of −8.7 ± 0.6 mV.

*Py*MSP1/8-sec and *Py*MSP1/8-mem mRNA were transcribed in vitro, replacing UTP with N1-methylpseudouridine-5′-triphosphate, and then capped and purified. A construct encoding *Xenopus* elongation factor 1α (Xef) served as a positive control in all transcription reactions. As shown in Figure 2A, intact transcripts of the correct size (Xef: 1890 bp; *Py*MSP1/8-sec: 2021 bp; *Py*MSP1/8-mem: 2116 bp) were visualized by agarose gel electrophoresis. To examine protein expression in mammalian cells, 1 µg of capped and purified *Py*MSP1/8-sec, *Py*MSP1/8-mem, and Xef mRNA was transfected into CHO-K1 cells, and incubation continued for 24, 48, and 72 h. Maximum protein expression for both *Py*MSP1/8-sec and *Py*MSP1/8-mem was observed at 48 h post transfection, and subsequent analysis focused on this time point. Post transfection, cell culture supernatant and cell pellets were examined by immunoblot analysis using a polyclonal rabbit anti-*Py*MSP8 serum. As shown in Figure 2B, *Py*MSP1/8-sec migrated as a 67–68 kDa doublet, predominantly detected in the cell culture supernatant, indicative of proper secretion. Migration of *Py*MSP1/8-sec was slightly higher than its predicted molecular weight, likely due to the presence of albumin in the cell culture media migrating in the region of 66 kDa, creating some distortion. The possibility of minor post-translational modification cannot be excluded. In contrast to *Py*MSP1/8-sec, the majority of the *Py*MSP1/8-mem product remained cell-associated, with only a minor fraction being released into the culture supernatant. This suggests that *Py*MSP1/8-mem was properly anchored on the plasma membrane. As expected, sero-reactive proteins were not detected in the cell-associated or supernatant fractions of Xef mRNA-transfected cells.

**Figure 3:**
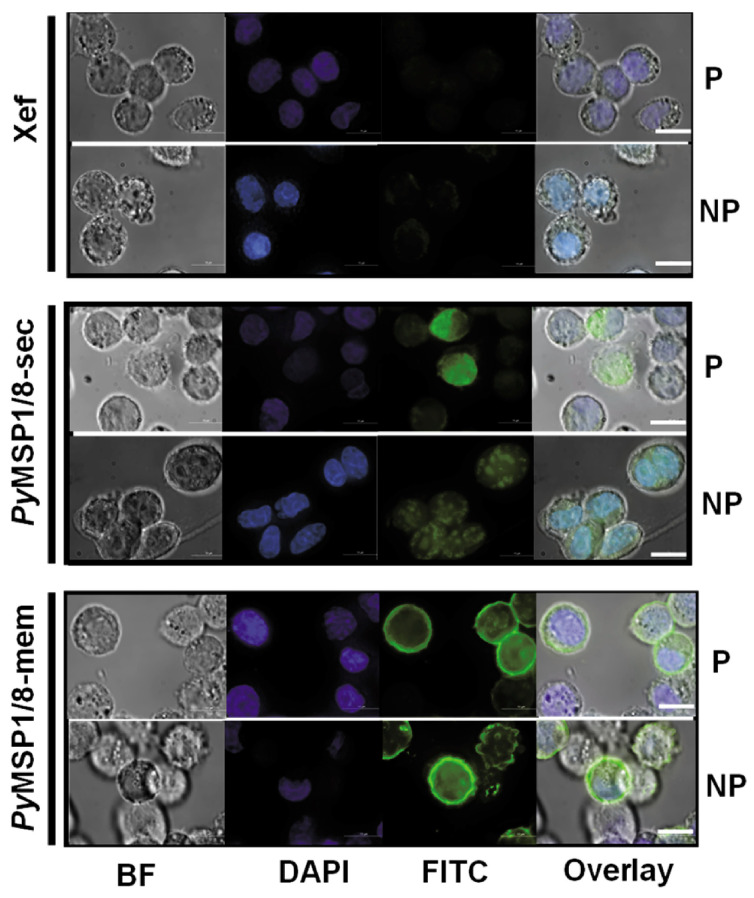
GPI-anchored *Py*MSP1/8-mem is expressed on the surface of transfected CHO cells. Cells were grown in four-well chamber slides and transfected with 1 µg/well of capped, purified Xef (top panels), *Py*MSP1/8-sec (middle panels), or *Py*MSP1/8-mem (bottom panels) mRNA according to manufacturer’s protocol and assayed at 48 h. Cells were fixed, a subset was permeabilized with Triton X-100, and immunofluorescence was performed using rabbit anti-*Py*MSP8 sera and a FITC-labeled secondary antibody. “P” indicates permeabilized cells; “NP” indicates non-permeabilized cells. Brightfield (BF), DAPI (blue), FITC (green), and overlay are shown. Scale bar 10 µm.

To confirm surface localization, expression of *Py*MSP1/8-mem was assessed by immunofluorescence assay. CHO K1 cells were transfected with 1 µg capped and purified Xef, *Py*MSP1/8-sec, or *Py*MSP1/8-mem mRNA. At 48 h post transfection, cells were fixed or fixed and permeabilized, and *Py*MSP1/8 localization was determined using rabbit anti-*Py*MSP8 sera. As shown in Figure 3, *Py*MSP1/8-mem was readily detected on the surface of fixed, non-permeabilized transfected cells, confirming surface expression. In contrast, *Py*MSP1/8-sec was only detected in transfected cells that were fixed and permeabilized with Triton X-100. No fluorescence of Xef mRNA-transfected cells was observed. Combined, these data indicate that mRNAs encoding *Py*MSP1/8-sec and *Py*MSP1/8-mem vaccine antigens were appropriately translated in mammalian cells and either secreted or surface membrane-anchored as targeted.

### 3.2. The PyMSP1/8-Sec mRNA Vaccine Is Potently Immunogenic and Protective Against Lethal Blood-Stage Malaria, Superior to a Recombinant Antigen/Adjuvant Formulation

The immunogenicity and efficacy of *Py*MSP1/8 mRNA vaccines were compared to a recombinant *Py*MSP1/8 antigen plus adjuvant vaccine, previously shown to protect against lethal *P. yoelii* 17XL blood-stage malaria [43]. For delivery, *Py*MSP1/8-sec, *Py*MSP1/8-mem, and Xef mRNAs were encapsulated into lipid nanoparticles (95–97% efficiency). Following membrane extrusion, LNPs were shown to be of uniform size (100–110 nm) by dynamic light scattering analysis (Figure 2C). To begin, outbred CD1 mice (*n* = 10 per group; 5 female, 5 male) were immunized i.m. at a 3-week interval with *Py*MSP1/8-sec mRNA/LNPs at 5 µg per dose. Control mice received a similarly produced mRNA/LNP encoding Xef. For comparison, groups of mice (*n* = 10 per group; 5 female, 5 male) were immunized s.c. in parallel with recombinant *Py*MSP1/8 (5 µg per dose) formulated with Quil A as adjuvant or with adjuvant alone. Small volumes of sera were collected following each immunization to determine antibody titers to the *Py*MSP1_19_ and *Py*MSP8 domains by ELISA.

**Figure 4:**
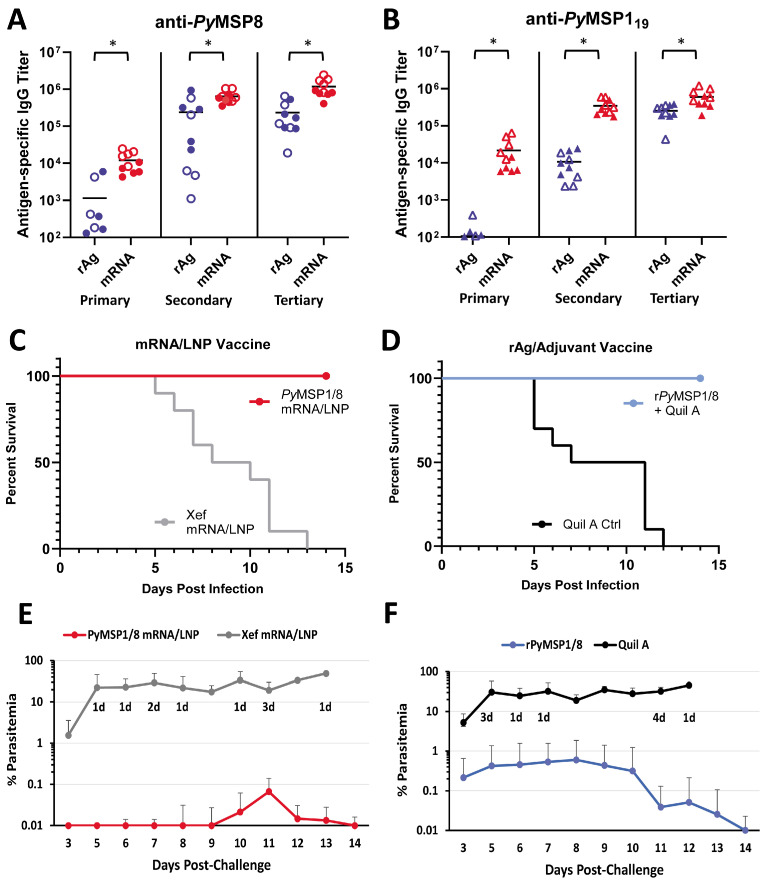
Immunogenicity and efficacy of recombinant antigen versus *Py*MSP1/8-sec mRNA/LNP vaccines. *Py*MSP8-specific (**A**) and *Py*MSP1_19_-specific (**B**) IgG titers in mice immunized three times with recombinant antigen (blue) or *Py*MSP1/8-sec mRNA/LNP (red) vaccines were determined by ELISA. Male mice are indicated by closed circles (**A**) or closed triangles (**B**); female mice are indicated by open circles (**A**) or open triangles (**B**). Asterisks indicate statistically significant differences (Mann–Whitney test; *p* < 0.01). Mice immunized with *Py*MSP1/8-sec mRNA/LNP (red; (**C**,**E**)) or recombinant antigen (blue; (**D**,**F**)) vaccines or respective controls were challenged with *P. yoelii* 17XL pRBCs. Percent survival (**C**,**D**) and percent blood parasitemia (**E**,**F**) were monitored daily. “d” refers to the number of deceased animals at each time point.

As shown in Figure 4A,B, the *Py*MSP1/8-sec mRNA/LNP vaccine induced high titers of antibodies to both vaccine components, exceeding titers induced by the recombinant antigen plus adjuvant formulation. After a single immunization, anti-*Py*MSP8 IgG titers in *Py*MSP1/8-sec mRNA/LNP-immunized mice were 10-fold higher than in r*Py*MSP1/8-immunized mice (11,975 ± 7066 vs. 1146 ± 2110, *p* < 0.01). Differences in the antibody response to the *Py*MSP1_19_ domain after a single immunization were more pronounced, with a 200-fold difference in titer (21,708 ± 20,521 vs. 107 ± 109, *p* < 0.01). Anti-*Py*MSP1_19_ antibody responses were significantly boosted by a second immunization (16-fold, *p* < 0.01) and third immunization (1.7-fold, *p* < 0.01) with *Py*MSP1/8-sec mRNA/LNPs. As expected, anti-*Py*MSP1_19_ antibody responses were also boosted by a second immunization (100-fold, *p* < 0.01) and third immunization (24-fold, *p* < 0.01) with the r*Py*MSP1/8 formulation. Nevertheless, anti-*Py*MSP1_19_ IgG titers remained significantly higher in *Py*MSP1/8-sec mRNA/LNP-immunized animals relative to the r*Py*MSP1/8-immunized group following both the second and third immunizations (*p* < 0.01). The final pre-challenge anti-*Py*MSP1_19_ IgG titers were 602,981 ± 317,174 and 255,177 ± 105,165 in *Py*MSP1/8-sec mRNA/LNP- versus r*Py*MSP1/8-immunized mice, highlighting the enhanced immunogenicity of the mRNA/LNP vaccine. Interestingly, the final anti-*Py*MSP1_19_ IgG and anti-*Py*MSP8 IgG titers were approximately 2-fold higher in female vs. male mice immunized with *Py*MSP1/8-sec mRNA/LNPs (*p* < 0.05). These sex differences were not observed mice immunized with r*Py*MSP1/8 in adjuvant. Immunization with the Xef mRNA/LNPs or Quil A alone did not induce anti-*Py*MSP1_19_ or anti-*Py*MSP8 antibody responses above background. Anti-*Py*MSP1/8 IgG subclass analysis (IgG1 and IgG2a/c) was also evaluated by ELISA in animals immunized with *Py*MSP1/8-sec and r*Py*MSP1/8 (Figure S3). Both immunization groups had a balanced Th1- vs. Th2-associated profile of antibodies, with no significant differences seen between groups in the ratio of IgG1 to IgG2a/c.

Two weeks following the third immunization, all mice were challenged by i.p. injection of 1 × 10^6^ lethal *P. yoelii* 17XL-parasitized RBCs. Thin tail-blood smears were prepared, and parasitemia and mortality were monitored through day 18 of *P. yoelii* infection. The *Py*MSP1/8-sec mRNA/LNP vaccine (Figure 4C,E) was potently protective, with all *Py*MSP1/8-sec mRNA/LNP-immunized mice surviving an otherwise lethal challenge infection. Remarkably, 5 out of 10 *Py*MSP1/8-sec mRNA/LNP-immunized mice exhibited no detectable *P. yoelii* pRBCs in circulation (LOD 0.01%). Furthermore, the maximum parasitemia in the remaining five *Py*MSP1/8-sec mRNA/LNP-immunized mice did not exceed 0.5%. Consistent with prior studies, immunization with the r*Py*MSP1/8 formulated with Quil A was also protective, with 100% survival (Figure 4D). However, 60% of animals immunized with the r*Py*MSP1/8 vaccine developed parasitemia between 0.1% and 3.5% before clearing the infection. In animals that became infected, the mean peak parasitemia in *Py*MSP1/8-sec mRNA/LNP-immunized mice was significantly lower than the mean peak parasitemia in the r*Py*MSP1/8 vaccine group (*p* < 0.05). The *P. yoelii* 17XL infection was lethal in all Xef mRNA/LNP and adjuvant control mice. Combined, the immunogenicity and efficacy of the *Py*MSP1/8-sec mRNA/LNP vaccine was robust and superior to a comparable recombinant antigen formulation previously shown to protect against lethal blood-stage malaria.

### 3.3. The Immunogenicity and Efficacy of the PyMSP1/8-Mem mRNA Vaccine Are Superior to the PyMSP1/8-Sec mRNA Vaccine When Administered at Low Dose

**Figure 5:**
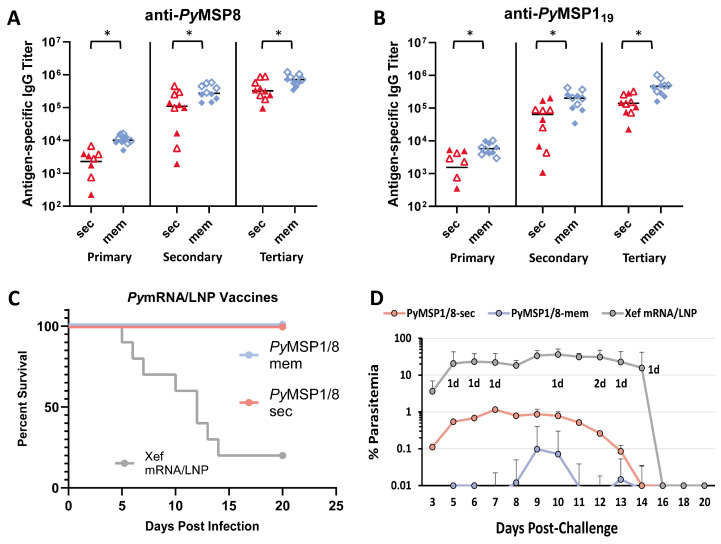
Immunogenicity and efficacy of *Py*MSP1/8-sec versus *Py*MSP1/8-mem vaccines. *Py*MSP8- (**A**) and *Py*MSP1_19_-specific (**B**) IgG titers in mice immunized three times with *Py*MSP1/8-sec (red) or *Py*MSP1/8-mem (blue) mRNA/LNP vaccines (1 µg/dose) were determined by ELISA. Male mice are indicated by closed triangles or closed diamonds; female mice are indicated by open triangles or open diamonds. Asterisks indicate statistically significant differences (Mann–Whitney test; *p* < 0.01). (**C**,**D**) Mice were immunized with either *Py*MSP1/8-sec (pink), *Py*MSP1/8-mem (blue), or Xef control (gray) vaccines and challenged with *P. yoelii* 17XL-parasitized RBCs. Percent survival (**C**) and percent parasitemia (**D**) were monitored daily. “d” refers to the number of deceased animals at each time point.

Next, the immunogenicity and efficacy of *Py*MSP1/8-sec versus *Py*MSP1/8-mem mRNA/LNP vaccines were compared. To increase the ability to detect differences between the two vaccines, the immunization dose was reduced from 5 µg to 2.5 µg. At this lowered dose, the efficacy against lethal *P. yoelii* 17XL challenge infection elicited by both mRNA/LNP vaccines was high and not significantly different. Subsequently, outbred CD1 mice (*n* = 10 per group; 5 female, 5 male) were immunized and boosted twice at a 3-week interval with *Py*MSP1/8-sec or *Py*MSP1/8-mem mRNA/LNP vaccines administered at 1 µg per dose. Control mice received Xef mRNA/LNPs. As shown in Figure 5A,B, both mRNA/LNP vaccines induced high titers of antibodies to *Py*MSP8 and *Py*MSP1_19_. Notably, the membrane-anchored *Py*MSP1/8 elicited significantly higher titers of anti-*Py*MSP8 and anti-*Py*MSP1_19_ IgG compared to secreted *Py*MSP1/8 after each of the three immunizations (*p* < 0.02). After the full series of immunizations, the mean anti-*Py*MSP8 titer was 726,669 ± 262,686 in the *Py*MSP1/8-mem-immunized group compared to 415,290 ± 279,445 in *Py*MSP1/8-sec-immunized animals (*p* < 0.05). Similarly, the final mean *Py*MSP1_19_ titer was 472,068 ± 265,110 in the *Py*MSP1/8-mem-immunized group compared to 158,020 ± 99,548 in *Py*MSP1/8-sec-immunized animals (*p* < 0.01). Overall, the final antibody titers were again 1.5–2 fold higher in female vs. male mice, with significant differences noted in *Py*MSP1/8-mem-immunized mice (anti-*Py*MSP8, *p* = 0.016; anti-*Py*MSP1_19_, *p* = 0.055).

Two weeks following the third immunization, all mice were challenged by i.p. injection of 1 × 10^6^ lethal *P. yoelii* 17XL-parasitized RBCs. As shown in Figure 5C, low-dose immunization with both *Py*MSP1/8-mem and *Py*MSP1/8-sec mRNA/LNP vaccines afforded high and significant protection, with all immunized animals clearing an otherwise lethal blood-stage infection. However, comparing the parasite burden in *Py*MSP1/8-mem- versus *Py*MSP1/8-sec mRNA/LNP-immunized animals, significant differences were observed (Figure 5D). First, no circulating blood-stage parasites were detected in a higher proportion of *Py*MSP1/8-mem-immunized animals relative to *Py*MSP1/8-sec-immunized animals (70% vs. 30%, *p* < 0.05, LOD 0.01%). Second, mean peak parasitemia in *Py*MSP1/8-mem-immunized animals was significantly lower than in *Py*MSP1/8-sec-immunized mice (0.11 ± 0.30% vs. 1.63% ± 1.45%). Combined, these data demonstrate that *Py*MSP1/8 mRNA/LNP vaccines remain highly efficacious at low dose and that targeting *Py*MSP1/8 to be membrane-anchored and surface-displayed improves both vaccine immunogenicity and efficacy in comparison to secreted *Py*MSP1/8.

## 4. Discussion

Advances in mRNA/LNP vaccine technology and the successful deployment of COVID-19 mRNA vaccines worldwide highlight the significant advantages of this platform, of which high vaccine immunogenicity and efficacy are most notable. From a vaccine development perspective, it is also encouraging that preclinical studies of mRNA/LNP vaccines in mice readily translated to subsequent trials in nonhuman primates and then human subjects [49,50]. The Spike-targeted mRNA/LNP vaccines for SARS-CoV-2 demonstrated safety, immunogenicity, and efficacy in adults, adolescents, young children, and infants [17,51,52,53,54]. However, there are questions regarding the applicability of this platform for malaria vaccines. The primary goal of this study was to evaluate the suitability of the mRNA/LNP platform in vivo, specifically for blood-stage malaria vaccine development. We previously demonstrated that a model chimeric, recombinant antigen vaccine based on *Py*MSP1_19_ fused to full-length *Py*MSP8 potently protected against lethal *P. yoelii* 17XL malaria in mice. With this vaccine, *Py*MSP8-specific T cells provided help to induce and maintain high levels of protective, merozoite-neutralizing antibodies [43,48]. Similarly, we have also demonstrated that *Pf*MSP8 is an effective carrier protein for several *P. falciparum* vaccine candidates, including *Pf*CSP, *Pf*MSP1_19_, *Pf*MSP2, and Pfs25 [42,55,56,57].

We compared the r*Py*MSP1/8 vaccine formulated with Quil A as adjuvant with an mRNA/LNP vaccine encoding the same chimeric *Py*MSP1/8 antigen, designed to be secreted from host cells. The *Py*MSP1/8 mRNA/LNP vaccine was robust, impressively inducing significantly higher antibody titers than the recombinant antigen/adjuvant vaccine, resulting in enhanced protection from an otherwise lethal blood-stage infection. There have been a few studies directly comparing recombinant antigen plus adjuvant vaccines versus mRNA/LNP vaccines. For SARS-CoV-2 spike protein receptor binding domain (RBD), immunization of mice with an mRNA/LNP construct induced a balanced IgG1/IgG2a response, whereas the recombinant antigen plus adjuvant formulation induced a more prominent IgG1/Th2-biased response. Although these two vaccines induced similar titers of antigen-specific IgG, the mRNA/LNP formulation induced a stronger IFNγ-secreting, antigen-specific T-cell response [58]. In a second study, Guo et al. [59] compared the immunogenicity of several different vaccine platforms, including a whole inactivated virus, recombinant protein, mRNA/LNPs and a nanoparticle-based vaccine targeting the SARS-CoV-2 RBD. Antibody titers and germinal center formation were superior in the nanoparticle and mRNA/LNP platforms compared to the recombinant or whole inactivated vaccine. In a comparison of recombinant protein, DNA, and mRNA/LNP platforms for the merozoite vaccine candidate *Pf*GBP130, only antibodies induced by the nucleic acid-based vaccines were capable of inhibiting blood-stage parasite growth in vitro [60]. In our study, the immunogenicity and in vivo efficacy of the *Py*MSP1/8-sec mRNA/LNP vaccine was superior to that of r*Py*MSP1/8 formulated with Quil A. We recognize that interpretation of results from side-by-side comparisons of recombinant antigen-based vaccines versus mRNA/LNP vaccines is complicated. Improved performance of an mRNA/LNP vaccine could depend on increased effective antigen dose due to high and/or sustained in vivo antigen expression, protein folding that more closely mimics a native conformation, improved uptake and processing by APCs, or a combination of the above. Notwithstanding these caveats, our data clearly demonstrate that the mRNA/LNP platform is very effective for a blood-stage malaria vaccine in which protection depends on the induction of merozoite neutralizing antibodies that recognize the disulfide-bond-dependent, double-EGF-like domains of *Py*MSP1_19_ and *Py*MSP8.

For mRNA/LNP vaccines, the cell- and tissue-specific expression of vaccine targets along with the kinetics of expression, processing, and presentation of the antigen in vivo can influence immunogenicity and overall efficacy. For soluble, secreted vaccine candidates, APCs can endocytose exogenous antigen for presentation on MHC class II to CD4+ T cells. In contrast, vaccine candidates targeted for cytosolic expression can utilize the endogenous antigen processing machinery to present peptides complexed with MHC class I to CD8+ T cells. Proteins anchored to the surface membrane of host cells may be accessible to B cells for an extended period of time and/or be more efficiently delivered to a cross-presentation pathway driving both CD4+ and CD8+ T-cell responses. Straightforward modifications to the design of our *Py*MSP1/8 mRNA/LNP vaccine construct allowed us to begin to evaluate the impact of subcellular localization of *Py*MSP1/8 expression on immunogenicity and efficacy. We confirmed that a mouse MHC class II signal sequence effectively directed secretion of the *Py*MSP1/8-sec from host cells and that addition of the C-terminal GPI anchor signal sequence from mouse CD14 targeted *Py*MSP1/8-mem for surface display on host cells. Most notably, our results demonstrate that the surface-expressed *Py*MSP1/8-mem vaccine outperformed the secreted version, inducing significantly higher antibody titers against both *Py*MSP8 and *Py*MSP1_19_ domains at a low immunization dose (1 µg). This led to improved protection against blood-stage malaria considering host survival, maximum parasite burden, and the number of animals remaining parasite-free.

In the present immunogenicity study involving outbred mice of both sexes, we noted a modest but significant increase in the magnitude of the antibody response in female vs. male mice immunized with mRNA/LNP vaccines. We did not see this difference in mice immunized with recombinant antigen formulated with Quil A in this study or in prior studies with various recombinant vaccine candidate antigens formulated with Alum or GLA-SE as adjuvant [55,56,57]. Certainly, sex differences in the immune response elicited upon infection with a variety of pathogens and/or in response to different types of vaccines (attenuated or subunit) have been reported [61]. Differences in the complement of sex chromosomes and associated genes as well as sex hormone levels have been implicated [62]. Data on sex bias upon administration of mRNA vaccines considering immunogenicity, safety, and efficacy are variable, with no clear consensus [63,64,65]. While noting the modest sex bias in antigen-specific antibody titers in our *Py*MSP1/8 mRNA/LNP-vaccinated mice, female and male mice were still both solidly protected against blood-stage malaria. As studies with *P. falciparum* mRNA vaccines progress, sex as a variable in response to vaccination will be of interest.

In addition to vaccines for a variety of viral and bacterial pathogens, the evaluation of the mRNA/LNP platform for vaccines targeting parasitic diseases including malaria has begun. The results are encouraging. mRNA-based vaccines for malaria involving *Pf*CSP, *Pf*CelTOS, and *Pf*s25 [21,22,23,24]; one limited study involving *Pf*GARP [25]; and one study with a Venezuelan equine encephalitis (VEE) self-amplifying RNA replicon for *Pf*Rh5 [26] have been reported. The design of the mRNA constructs tested have primarily targeted the respective candidate antigens for secretion. Immunogenicity has been demonstrated with over a range of vaccine doses, with the functionality of antibodies nicely demonstrated using in vitro assays of parasite neutralization or upon challenge infection with transgenic rodent malaria sporozoites expressing *Pf*CSP. In a recent study, Scaria et al. [30] evaluated mRNA/LNP vaccine constructs focused on *P. falciparum* transmission blocking candidates *Pf*s25 and *Pf*s240D1, testing various signal peptides, transmembrane domains, and GPI anchor signal sequences. In mouse immunogenicity studies using single and combined antigen formulations, antibody responses were higher following immunization with antigens containing a GPI anchor or TM domain, as was the ability of these antibodies to reduce transmission in a standard membrane feeding assay. Similar to *Pf*s25, the immunogenicity of mRNA/LNP vaccines targeting the 25 kDa sexual-stage antigen of *P. vivax* induced higher levels of memory B cells and CD4+ T cells when compared to recombinant antigen formulations, with better durability of responses across a 7-month follow-up period [66]. Constructs that targeted *Pv*s25 to be GPI-anchored to host membranes were preferred over secreted *Pv*s25. In a study focused on the hookworm *Necatur americanus* [67], mRNA vaccines were designed to encode glutathione S-transferase-1 (*Na*-GST-1) expressed as cytosolic, secreted, or plasma-membrane-bound antigens and with immunogenicity compared to recombinant *Na*-GST-1. mRNA constructs induced higher antibody titers than the recombinant antigen formulation, with the membrane-anchored *Na*-GST inducing higher antibody titers compared to cytosolic and secreted constructs. Of interest, the cytosolic antigen induced superior CD8+ T-cell responses, indicating that cellular localization can indeed drive varying arms of the immune response.

To our knowledge, ours is the first study examining mRNA/LNP vaccines targeting blood-stage malaria parasites in which potent, in vivo efficacy was demonstrated. We report successful design, expression, and subcellular localization of the chimeric model antigen *Py*MSP1/8 from two constructs. These mRNA/LNP vaccines were highly immunogenic and potently protective. Looking forward, the effectiveness of these blood-stage mRNA/LNP vaccines at relatively low dose is very attractive when considering the need to incorporate multiple plasmodial proteins into a single multivalent formulation. We strongly believe that it will be essential to include a blood-stage component in such a multivalent vaccine to reduce clinical disease and now demonstrate the potential of the mRNA/LNP platform to achieve this goal. With this mRNA/LNP platform, opportunities to modify constructs and/or immunization protocols to concurrently drive both antibody-mediated and cell-mediated protective immune responses that persist can be exploited.

## Data Availability

The data generated from this study are graphically presented in the article/Supplementary Materials. The original data and individual data points for each of these figures are deposited in the NIH supported repository ImmPort (http://www.immport.org) (accessed on 20 June 2025), accession #SDY3126. Further inquiries can be directed to the corresponding author.

## Supplementary Materials

The following supporting information can be downloaded at https://www.mdpi.com/article/10.3390/vaccines13070702/s1. Figure S1: Characterization of *Py*MSP1/8 mRNA vaccines, original images; Figure S2: Immunofluorescence assay of *Py*MSP1/8 sec- and *Py*MSP1/8-mem mRNA-transfected CHO cells, original images; Figure S3: Antigen-specific IgG subclass analysis in *Py*MSP1/8-sec mRNA- and r*Py*MSP1/8-immunized mice.
