# Supplementary material for: An mRNA Vaccine Expressing Blood-Stage Malaria Antigens Induces Complete Protection Against Lethal Plasmodium yoelii
Source: Vaccines (Basel). 2025 Jun 28;13(7):702. doi: 10.3390/vaccines13070702 (PMC12300687; doi:10.3390/vaccines13070702)
Supplement: Supplementary file 1 [file vaccines-13-00702-s001.zip › vaccines-3670271-supplementary.pdf]

Supplemental Material

A mRNA vaccine expressing blood-stage malaria antigens induces complete protection against lethal *Plasmodium yoelii*

Amy C. Ott [1,*], Patrick J. Loll [2] and James M. Burns, Jr. [1]

[1] Center for Molecular Parasitology, Department of Microbiology and Immunology, Drexel University College of Medicine, 2900 West Queen Lane, Philadelphia PA 19129

[2] Department of Biochemistry and Molecular Biology, Drexel University College of Medicine, 215 North 15th St., Philadelphia PA, 19102

*****Correspondence: ac3482@drexel.edu; 215-991-8490

A – Figure 2A, main manuscript

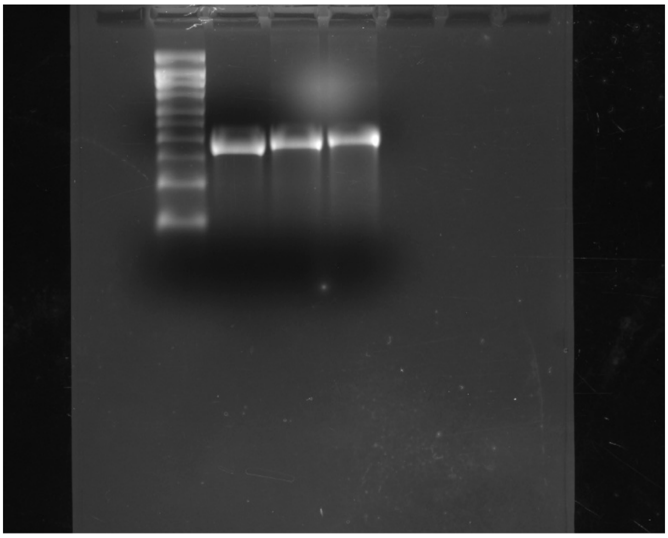

B – Figure 2B, main manuscript[t

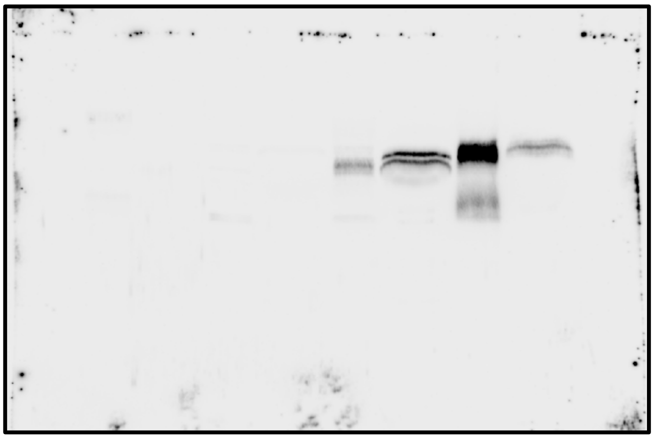

Figure S1. Characterization of PyMSP1/8 mRNA vaccines, original images. **(A)** Full images of Figure 2A – Ethidium bromide stained 1% formaldehyde agarose gel of in vitro transcribed Xef mRNA (lane 1) *Py*MSP1/8-sec mRNA (lane 2) and *Py*MSP1/8-mem mRNA. **(B)** Full image of Figure 2B immunoblot of CHO cell culture supernatant and cell pellets 48 hours after transfection with Xef, *Py*MSP1/8-sec, and *Py*MSP1/8-mem mRNA using polyclonal anti-*Py*MSP8 antisera.

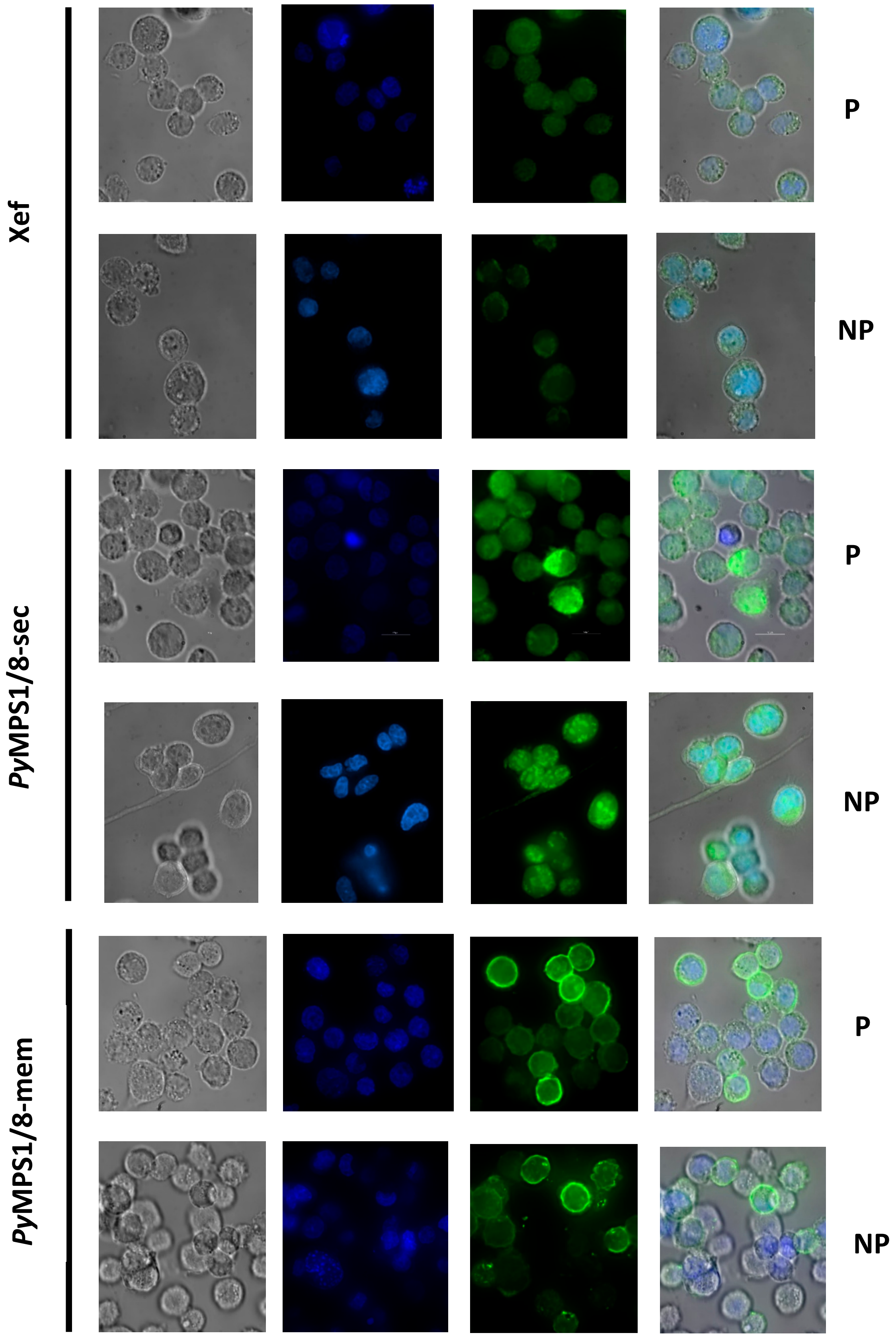
Xef
P
NP
PyMPS1/8-sec
P
NP
PyMPS1/8-mem
P
NP

Figure S2. GPI-anchored *Py*MSP1/8-mem is expressed on the surface of transfected CHO cells, original images. Original images of Figure 3 – cells were transfected with capped, purified Xef (top panels), *Py*MSP1/8-sec (middle panels), or *Py*MSP1/8-mem (bottom panels) and assayed at 48 hours. Cells were fixed, a subset was permeabilized and immunofluorescence was performed using rabbit anti-*Py*MSP8 sera and a FITC-labelled secondary antibody. “P” indicates permeabilized cells; “NP” indicates non-permeabilized cells. Brightfield (BF), DAPI, FITC, and overlay are shown.

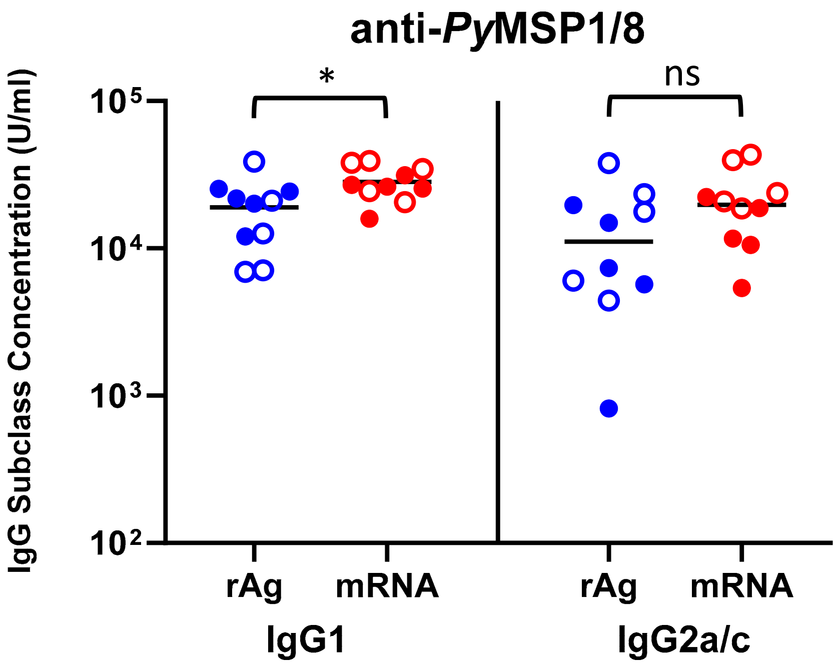

Figure S3. Antigen-specific IgG subclass analysis in *Py*MSP1/8-sec mRNA and r*Py*MSP1/8 immunized mice. Sera from mice immunized with r*Py*MSP1/8 formulated with Quil A as adjuvant (blue) or with the *Py*MSP1/8-sec mRNA/LNP vaccine (red) were analyzed by ELISA for the presence of anti-*Py*MSP1/8 antibodies of the IgG1 or IgG2a/c subclasses. Male mice are indicated by closed circles while female mice are indicated by open circles. Asterisks indicate statistically significant differences (Mann-Whitney test; $p<0.01$; ns, not significant).
